# Overexpression of *CsCaM3* Improves High Temperature Tolerance in Cucumber

**DOI:** 10.3389/fpls.2018.00797

**Published:** 2018-06-12

**Authors:** Bingwei Yu, Shuangshuang Yan, Huoyan Zhou, Riyue Dong, Jianjun Lei, Changming Chen, Bihao Cao

**Affiliations:** ^1^Department of Vegetable Science, College of Horticulture, South China Agricultural University, Guangzhou, China; ^2^Key Laboratory of Biology and Germplasm Enhancement of Horticultural Crops in South China, Ministry of Agriculture, Guangzhou, China

**Keywords:** *CsCaM3*, calmodulin, high temperature tolerance, genes expression, cucumber

## Abstract

High temperature (HT) stress affects the growth and production of cucumbers, but genetic resources with high heat tolerance are very scarce in this crop. Calmodulin (CaM) has been confirmed to be related to the regulation of HT stress resistance in plants. *CsCaM3*, a CaM gene, was isolated from cucumber inbred line “02-8.” Its expression was characterized in the present study. *CsCaM3* transcripts differed among the organs and tissues of cucumber plants and could be induced by HTs or abscisic acid, but not by salicylic acid. *CsCaM3* transcripts exhibited subcellular localization to the cytoplasm and nuclei of cells. Overexpression of *CsCaM3* in cucumber plants has the potential to improve their heat tolerance and protect against oxidative damage and photosynthesis system damage by regulating the expression of HT-responsive genes in plants, including chlorophyll catabolism-related genes under HT stress. Taken together, our results provide useful insights into stress tolerance in cucumber.

## Introduction

Plants often endure various abiotic stresses throughout their growth and development, including low and high temperatures, salinity, and drought. High temperatures (HTs) is a serious abiotic factor that not only causes the loss of yield and diminishes quality of crops, but also leads to direct damage of crop plants, including protein denaturation and aggregation as well as oxidative injuries to membrane lipids. Accordingly, enhancing the environmental stress tolerance of crops is very important for agriculture (Varshney et al., 2012); this objective can alleviate the harm caused by HT stress and reduce associated agricultural losses.

Plant calmodulins (CaMs) comprise a multigene family that has been reported in many species. CaM, a conserved, small acidic protein, is one of the best characterized Ca^2+^-sensors and has four EF-hand motifs, each of which is involved in binding Ca^2+^. After binding to Ca^2+^, conformational changes in CaM lead to interactions with different target CaM-binding proteins (CaMBPs), which comprise disparate protein family members that perform a diverse range of roles in the cell (Crivici and Ikura, 1995). CaMs is a central component that modulates many cellular processes across multifarious physiological functions that regulate downstream target protein activities via Ca^2+^/CaM complex-mediated processes (Kim et al., 2009), including adjustment of metabolism, phytohormone signaling, ion transportation, and transcriptional regulation (Snedden and Fromm, 2001; Yang and Poovaiah, 2003; Bouché et al., 2005).

Many reports have indicated that plant CaMs play important roles in the modulation of tolerance to different stress (Kim et al., 2009), such as cold stress (Doherty et al., 2009), drought stress (Wan et al., 2012), heat stress (Li et al., 2005; Li and Gong, 2009) and so on. For example, overexpression of *AtCaM3* in *Arabidopsis* increased heat tolerance (Zhang et al., 2009). In tobacco, CaM isoforms were confirmed to be related to regulating resistance to two fungal pathogens (*Rhizoctonia solani* and *Pythium aphanidermatum*) and one bacterial pathogen (*Ralstonia solanacearum*; Takabatake et al., 2007). *SCaM-*4 from soybean was significantly induced by the presence of a pathogen (Park et al., 2004) or NaCl stress (Park et al., 2009). The expression level of *OsCaM1* in rice was enhanced by NaCl, mannitol, or wounding, and *OsCaM3* was also induced by NaCl or damage (Phean-O-Pas et al., 2005). Ca^2+^ and CaM are also able to protect against oxidative harm under heat stress (Larkindale and Knight, 2002) and increase the heat tolerance of suspension-cultured tobacco cells (Li and Gong, 2009). Those reports clearly indicated that plant CaMs enhance stress-tolerance.

Cucumber (*Cucumis sativus* L.) is an important vegetable crop that is planted worldwide and originated from subtropical areas. As a thermophilic crop, the optimum temperature of cucumber growth is 25–30°C. Temperatures above 35°C can often lead to thermal damage to cucumber plants, and cucumber leaves and stems show wilt and dead tissue within a short time at temperatures of 50°C (Talanova et al., 2006). In China, cucumber is cultivated in open fields during the summer growing season, where cucumber plants usually face the combined stress of strong light and heat, or sharp temperature increases in greenhouses. Even if ventilation systems are used, it is impossible to prevent substantial heat accumulation in greenhouses. Consequently, cucumber growth is often hindered by excessive daytime temperatures. This influences normal growth, photosynthesis and fruit of cucumber plants seriously, causing premature aging, yield reduction, and diminished quality. The breeding of cucumbers for heat tolerance has been slow because of the scarcity of heat-tolerant genetic resources in the species, and in research of heat resistance in cucumber (Ding et al., 2016; Li et al., 2016a,b), in where few reports of transgenic CaM genes inserted into the cucumber genome for the purpose of increasing stress-tolerance. This study examined the overexpression of the CaM gene *CsCaM3* in cucumber in order to enhance the heat tolerance of cucumber and provide the foundation for further improvements in the tolerance of cucumber plants to heat stress.

## Materials and Methods

### Plant Material, Gene Isolation, Vector Construction, and Transformation

The heat-susceptible cucumber inbred line “02-8” produced by our laboratory was used for all experiments in this study. DNA sequences of the CaM gene were found in the NCBI database and Cucumber Genome Database^1^. A fragment of the *CsCaM3* gene was amplified by PCR with the P1 forward primer (5′-CTCTAGAATGGCGGATCAGCTCACCGA-3′) and P2 reverse primer (5′-CGGATCCCCTTAGCCATCATGACCTTAAC-3′); underlined sequences show the *Xba*I and *Bam*HI restriction sites.

Evolutionary analyses were conducted in MEGA6 (Tamura et al., 2013). The evolutionary history was inferred using the neighbor-joining method (Saitou and Nei, 1987). The optimal tree with a summed branch length of 0.02022066 is shown (next to the branches). The evolutionary distances were computed using the Poisson correction method (Zuckerkandl and Pauling, 1965) and are within the units of the number of amino acid substitutions per site. The analysis involved eight amino acid sequences. All positions containing gaps and missing data were eliminated. There were a total of 149 positions in the final dataset.

The expression vector pBI-*CsCaM3* (**Supplementary Figure S1**) with the CaMV35S promoter and *npt*II gene was constructed and transferred to the *Agrobacterium tumefaciens* strain EHA105 by the freeze–thaw method. The transformation of cucumber was conducted as described by Cao et al. (2011). The first filial generation of the transgenic plants (OE-1, OE-2, and OE-3) were used in all experiments conducted at the three-leaf stage. The identification of transgenic cucumber plants was by Southern and northern blots. The hybridization was conducted using a DNA labeling and detection kit (Boehringer Mannheim, Mannheim, Germany), and the dosage of DNA and total RNA were 10 μg and 12 μg, respectively. A fragment of the 35S promoter was PCR amplified using the 35S-F forward primer (5′-ATAGTGGGATTGTGCGTCA-3′) and 35S-R reverse primer (5′-GCACCTACAAATGCCATCA-3′) and was then used as a probe for Southern blotting. The resulting fragment of *CsCaM3* was used as the probe for northern blotting. The heat treatment conditions for all experiments were under 43 and 25°C during the day and night, respectively.

### qRT-PCR Analysis

The expression levels of *CsCaM3* were analyzed at the three-leaf stage, with three biological and technical replicates. From the tissues of first top leaf, stem, and root of cucumber inbred line “02-8” seedlings were extracted total RNA with TRIzol reagents (Invitrogen, Carlsbad, CA, United States) after 43°C treatment for 12 h, and RNA was extracted from the first top leaf of seedlings after 0, 6, 12, and 24 h of temperature and hormone treatments. Solutions of 5 μM abscisic acid (ABA) and 100 μM salicylic acid (SA) were sprayed onto the leaves of seedlings under a 43°C temperature treatment. RNA was converted into cDNA with a kit (Genstar, Beijing, China). All qRT-PCR analyses were conducted by gene-specific primers (**Supplementary Table S1**) and a SYBR Premix Ex Taq kit (TaKaRa, Dalian, China). The RT-PCR protocol consisted of one cycle at 94°C for 3 min; 28 cycles of 94°C for 1 min, 55°C for 45 s, and 72°C for 90 s; and one cycle of 72°C for 7 min. The 2^-Δ^Ct method (Livak and Schmittgen, 2001) was used to assess the relative gene expression.

### Subcellular Localization of *CsCaM3*

Amplification of *CsCaM3* was conducted by PCR with the sub-Cam3F forward primer (5′-ATGGCGGATCAGCTCACCGA-3′) and the sub-Cam3R reverse primer (5′-CTTGGCCATCATGACCTTCA-3′), which does not contain a stop codon. In order to control *CsCaM3*-GFP vectors with a CaMV 35S promoter, the PCR products were fused into the pEZS-NL-GFP vector in-frame with the sequence of green fluorescent protein (GFP). The vector was transferred into *A. tumefaciens* strain EHA105, and the leaf epidermis of *Nicotiana benthamiana* leaves were injected with the transformed EHA105 (Na et al., 2016). GFP fluorescence of the transformed plants was observed after two days in darkness using a laser scanning confocal microscope (Leica TCS SP2, Leica Microsystems, Wetzlar, Germany).

### *In Situ* Hybridization

The *CsCaM3* gene was selected for localizing mRNA in the leaf and root tissues of cucumbers grown under HT stress (i.e., 43°C). Digoxigenin-labeled RNA probes were hybridized to the cucumber tissues of young leaf and root as described by Ma et al. (2012). The gene fragment was labeled using the DIG RNA Labeling Kit (SP6/T7; Roche, Shanghai, China). The fragments of probes were amplified from cDNA using gene-specific primers including SP6 and T7 RNA polymerase-binding sites (*CsCaM3*-SP6, 5′-ATTTAGGTGACACTATAGAAGATGGCGGATCAGCTCACCG A-3′; *CsCaM3*-T7, 5′-TAATACGACTCACTATAGGGAGACC TTAGCCATCATGA CCTTAAC-3′).

### Chlorophyll Fluorescence and Chlorophyll Content Assays

The measurement of chlorophyll fluorescence under minimal fluorescence (*F*_o_) was measured without actinic light. Then, the leaf was exposed to a saturation pulse to measure maximal fluorescence (*F*_m_). The optimal quantum yield of photosystem II (PSII) (*F*_v_/*F*_m_, *F*_v_ = *F*_m_ -*F*_o_) was derived from measured *F*_o_ and *F*_m_ values, where *F*_v_ was the difference between *F*_o_ and *F*_m_. Actinic light was turned on after saturation pulse, and the fluorescent signals reached the steady state slowly with intermittent saturation pulses. *F*_s_ determined the steady-state fluorescence yield, and *F*_m_′ was the resulting light-adapted maximum fluorescence. In this process, photochemical quenching of open PSII was measured, revealing photo-protective non-photochemical quenching (NPQ) and other heat dissipation mechanisms. *qP* is the fraction of open PSII reaction centers, characterized as the coefficients of photochemical fluorescence quenching (Genty et al., 1989; van Kooten and Snel, 1990). Electron transport rate (ETR) and effective quantum yield of PSII (Φ_PSII_) are valuable measurements in various plant stress studies. The *F*_v_/*F*_m_, Φ_PSII_, NPQ, and *qP* values were determined from the same individual using the following equations: Φ_PSII_ = (*F*_m_′ -*F*_s_)/*F*_m_′, NPQ = (*F*_m_/*F*_m_′) - 1, *qP* = (*F*_m_′ -*F*_s_)/(*F*_m_′ -*F*_o_), and ETR = Φ_PSII_ × PFD (photon flux density) × 0.5. Images were obtained using a Chlorophyll fluorescence imager (IMAGING-PAM, WALZ, Germany). Total chlorophyll, chlorophyll a, and chlorophyll b contents were assessed using a spectrophotometric method (Porra et al., 1989).

### Determination of MDA Content and Enzyme Activity

The malondialdehyde (MDA) content and peroxidase (POD) activities were tested according to the method described by Velikova et al. (2000). Superoxide dismutase (SOD) activity was measured using nitro blue tetrazolium (NBT) methods (Attar et al., 2006). Catalase (CAT) activities were measured using the method described by Dong et al. (2014).

### Detection of H_2_O_2_, Superoxide, and ROS Accumulation

Accumulations of superoxide and hydrogen peroxide (H_2_O_2_) were visually analyzed with NBT and 3,3-diaminobenzidine (DAB) staining using the methods described by Driever et al. (2009). Measurement of reactive oxygen species (ROS) was conducted using 2′,7′-dichlorodihydrofluorescein diacetate (DCFH-DA) staining. Leaf epidermal strips were peeled from cucumber leaves without chemical treatment. The leaf epidermal strips were floated on a solution of 50 μM DCFH-DA (Sigma Chemicals, St. Louis, MO, United States) for 30 min before treatment. ROS accumulation was observed by fluorescence microscopy using a Leica MZ FL III (excitation, 450 ± 490 nm; barrier 520 ± 560 nm).

### Determination of Root Viability

The viability of root tissue was assessed using the fluorescein diacetate/propidium iodide (FDA/PI) staining mixture method as described by Pan et al. (2001). The tips of roots (about 2.5 cm in length) were gently incubated in a FDA/PI mixture (containing FDA 12.5 g mL^-1^ and PI 5.0 g mL^-1^) for 10 min and then washed with distilled water. Images were observed with a fluorescent microscope (Olympus BX41; Olympus Corporation, Tokyo, Japan) under blue light excitation (460–495 nm) and emission at 510 nm and analyzed using a photographic apparatus (Olympus CamediaC-5060; Olympus, Tokyo, Japan) and analySIS software (Soft Imaging Solutions GmbH, Münster, Germany).

### Measurement of Stomatal Size and Abscisic Acid Content

Stomata were measured as described previously (Zhu et al., 2016). ABA content was measured as previously described (Kara et al., 1997). Enzyme-linked immunosorbent assay kits and 96-well (12 × 8) plates used in this study were purchased from the Crop Chemical Control Center of China Agricultural University. All assays and measurements were conducted in triplicate.

## Results

### Sequence Analysis of *CsCaM3*

**FIGURE 1:**
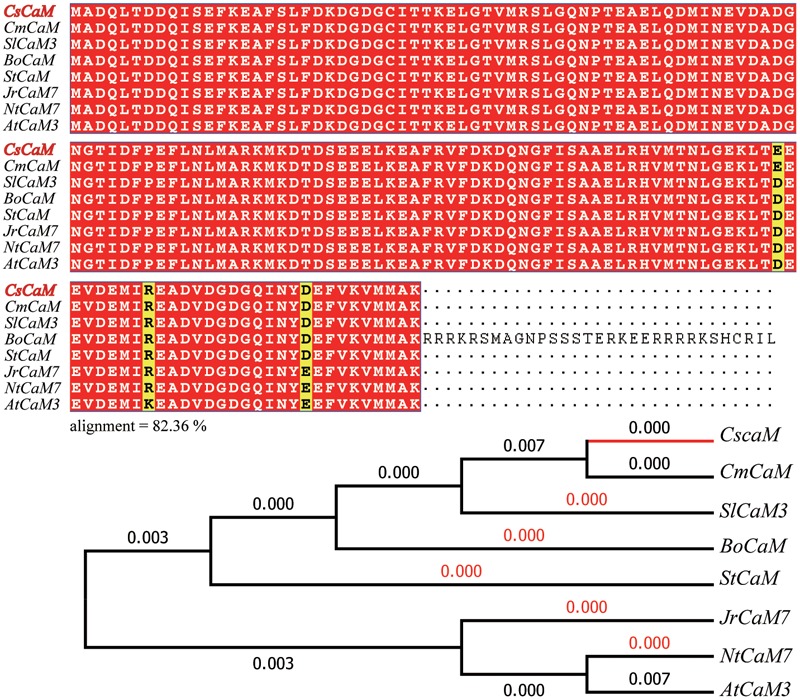
Sequence alignment and phylogenetic tree of *CsCaM3*. Alignment analysis of the inferred *CsCaM3* protein sequence with other inferred plant calmodulin proteins: *CsCaM* from *Cucumis sativus, CmCaM* from *Cucumis melo, AtCaM3* from *Arabidopsis thaliana, JrCaM7* from *Juglans regia, StCaM* from *Solanum tuberosum, NtCaM7* from *Nicotiana tabacum, BoCaM* from *Brassica oleracea*, and *SlCaM3* from *Solanum lycopersicum*. The numbers indicate branch lengths.

The NCBI database and Cucumber Genome Database (see text footnote 1) were searched to find DNA sequences of *CaM* genes. From these sequences, primers were designed to PCR amplify the *CaM* gene *CsCaM3* from cucumber total cDNA, resulting in the isolation of *CsCaM3*, a 450-bp ORF encoding 149 amino acids. The amino acid sequence of *CsCaM3* showed high similarity to *CsCaM* (XM_011655459.1) from cucumber, *CmCaM* (XM_008466393) from melon, *AtCaM3* AY091301) from *Arabidopsis, JrCaM7* (XM_018967153.1) from *Juglans regia, StCaM* (JX576246.1) from *Solanum tuberosum, NtCaM7* (XM_016640697.1) from *Nicotiana tabacum, BoCaM* (XM_013776345) from *Brassica oleracea*, and *SlCaM3* (NM_001321494) from *Solanum lycopersicum* (**Figure 1**).

### Expression Pattern and Subcellular Localization of *CsCaM3*

**FIGURE 2:**
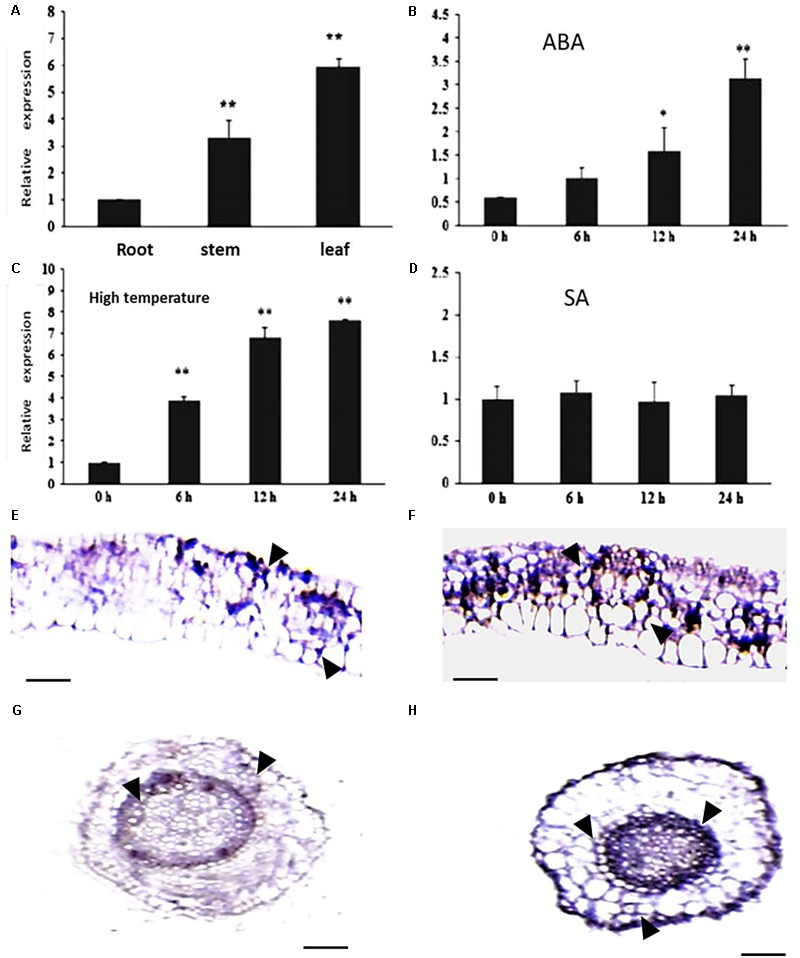
Analysis of expression characterization of *CsCaM3* and *in situ* hybridization. **(A)** The expression of *CsCaM3* in stem, leaf, and root tissues from cucumber inbred line “02-8.” **(B)** The expression of *CsCaM3* in leaf tissue after 5 μM ABA treatment at different times. **(C)** The expression of *CsCaM3* in leaf tissue after 43°C HT treatment at different times. **(D)** The expression of *CsCaM3* in leaf tissue after 100 μM SA treatment at different times. **(E,F)** *In situ* hybridization of *CsCaM*3 in leaf tissue in wild-type cucumber control plants and under 43°C HT treatments for 6 h, respectively. Scale bars = 100 μm. The arrows indicate hybridization signals. **(G,H)** *In situ* hybridization of *CsCaM3* in root tissue in wild-type cucumber control plants and under 43°C HT treatment for 6 h, respectively. ^∗^*P* < 0.05 and ^∗∗^*P* < 0.01 in comparisons of each time points. Scale bars = 50 μm. Each treatment was repeated three times.

**FIGURE 3:**
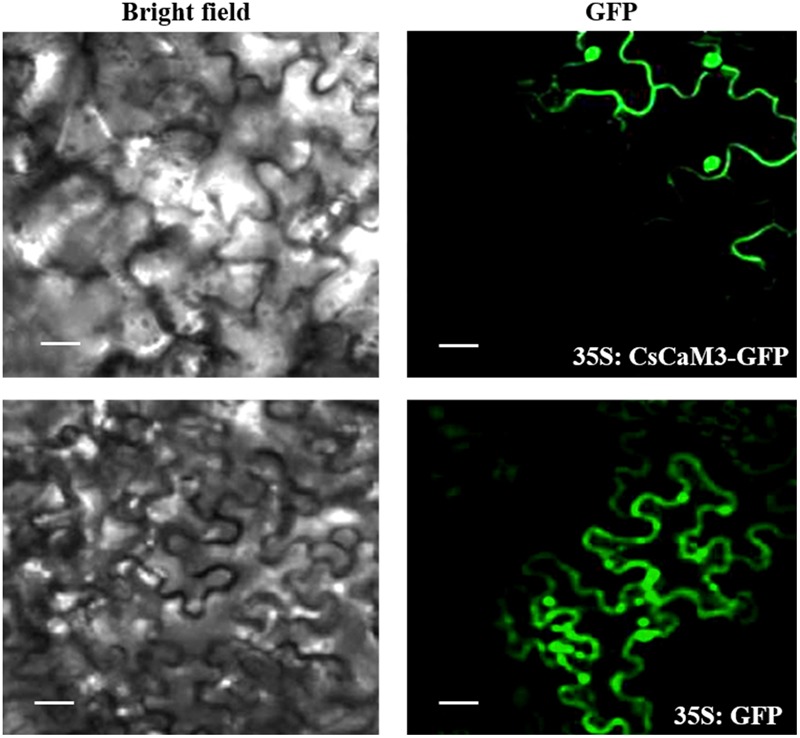
Subcellular localization of *CsCaM3*. The PCR products were fused with the pEZS-NL-GFP vector in-frame with the green fluorescent protein (GFP) sequence, and the CaMV 35S promoter controlled the *CsCaM3*-GFP vectors upstream. Subcellular distribution pattern of 35S:CsCaM3-GFP showing a nuclear and cell membrane distribution pattern at 24 h after infiltration into tobacco leaf epidermis tissues. Images of either dark field (green fluorescence) or bright field modes were acquired by fluorescence microscopy. Scale bars = 20 μm.

The expression pattern of *CsCaM3* was assessed in root, stem, and leaf tissues from cucumber inbred line “02-8.” The expression level of the gene was highest in leaves and lowest in roots (**Figure 2A**). ABA and HT stress treatment increased the expression of *CsCaM3*, but SA did not (**Figures 2B–D**). These results concurred with the results of situ hybridization of tissues was observed in leaf and root tissues, and the hybridization signals subjected to 43°C high-temperature stress treatment were stronger than that in the control group (**Figures 2E–H**). Subcellular location results indicated that CsCaM3 proteins are located in the cell membrane and nuclei (**Figure 3**). Taken together, *CsCaM3* is likely involved in the regulation of HT stress tolerance in cucumber.

### Overexpression of *CsCaM3* Increases the HT Stress Tolerance of Cucumber

**FIGURE 4:**
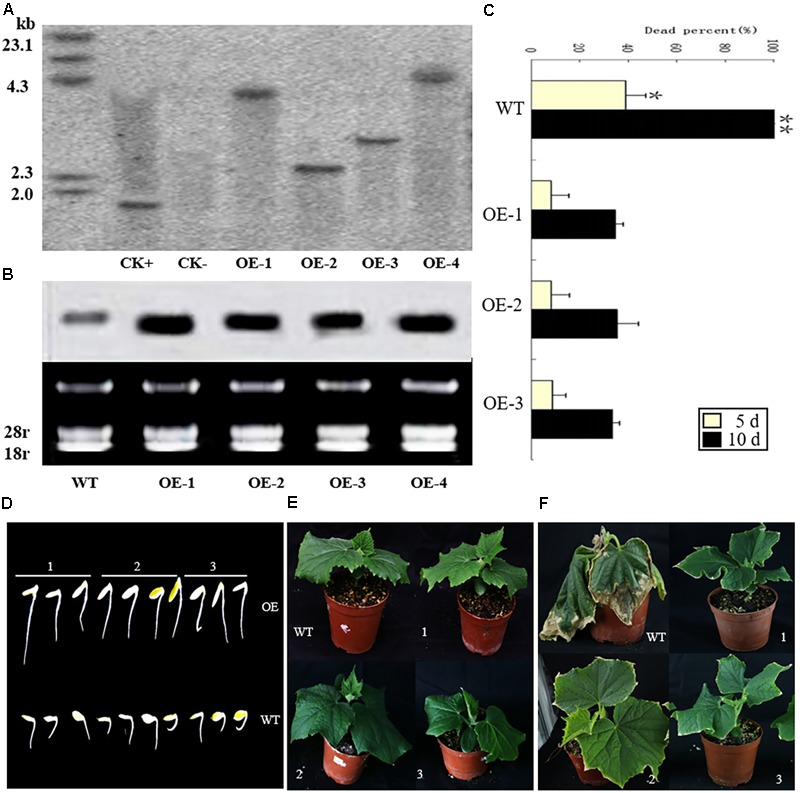
Identification of transgenic cucumber plants with Southern and northern blots. Heat-tolerance of wild-type (WT) and transgenic cucumber plants under 43 and 25°C day and night conditions, respectively. **(A)** Genomic DNA from transgenic Km-resistant plants was digested with *Hin*dIII, and a *NPT-II* gene fragment was used as a probe: CK+, the recombinant plasmid overexpressing *CsCaM*; CK–, the WT plants; OE-1, OE-2, OE-3, and OE-4, the transgenic plants. **(B)** Northern blots of the WT and transgenic plants. **(C)** Percentage of dead plants among the transgenic and WT plants under the HT treatment for 5 and 10 days. OE-1, OE-2, and OE-3 represent the T_1_ transgenic plants. There were more than 30 seedlings for each variety (WT, OE1, OE2, OE3). ^∗^*P* < 0.05 and ^∗∗^*P* < 0.01 in comparisons of WT and transgenic plants. **(D)** Germination of seeds from the T1 transgenic OE-1, OE-2, and OE-3 (1, 2, and 3, respectively) and WT lines under the 43°C HT treatment after 1 h on the first day. **(E)** The growth of T_1_ transgenic and WT plants under 25°C conditions. **(F)** Heat treatment symptoms of the T_1_ transgenic plants and WT plants under the HT treatments after 3 days.

In order to identify the function of *CsCaM3*, the *CsCaM3*-overexpression vector (i.e., *pBI-CsCaM3*) was constructed, from about 1000 explants used for transformation, four transgenic plants were obtained (**Supplementary Figure S2**), which were identified by genomic Southern and northern blotting (**Figures 4A,B**). The hybridization signal of *CsCaM3* in transgenic plants was determined to be stronger relative to the wild-type (WT) plants (**Figure 4B**). Three T1 transgenic plants (OE-1, OE-2, and OE-3) were obtained by selfing and used to assess heat-tolerance. The conditions of heat treatment were 43 and 25°C under day and night, respectively. More than 30 seedlings of each strains were used. After heat treatment for 5 days, more than 30% of WT plants were dead, but only 5% of the T1 transgenic plants were dead. After 10 days of HT treatment, all WT plants were dead, while about 60% of the T1 transgenic plants still survived (**Figures 4C,E,F**). The growth potential of transgenic plant radicles was stronger than that of WT plants (**Figure 4D**). Overexpression of *CsCaM3* may thus have improved the heat tolerance of cucumber plants.

### Determination of Root Viability and Chlorophyll Fluorescence of Transgenic Plants

**FIGURE 5:**
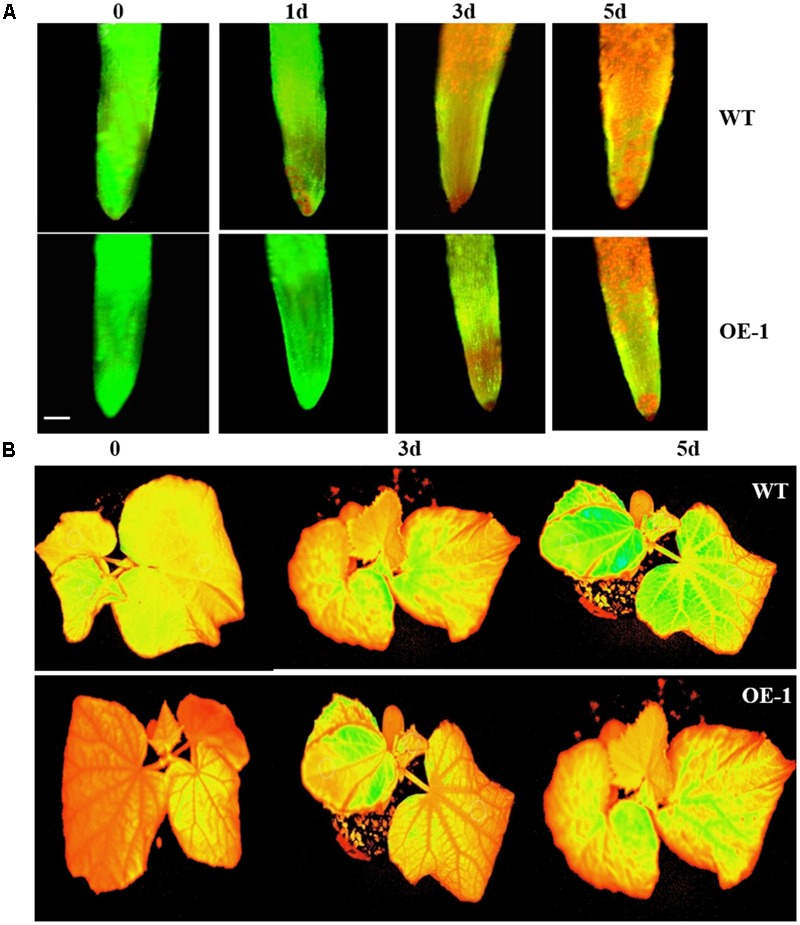
Viability of root cells and images of *Ft* (the instantaneous fluorescent signal) of cucumber plants under HT stress. **(A)** The viability of root cells of the cucumber plants under 43°C temperature treatment at different times. The yellow–green fluorescence indicates viable cells, while the red fluorescence indicates dead cells. Scale bars = 50 μm. **(B)** Images of *Ft* of cucumber plant under 43°C temperature treatment at different times. Green fluorescence indicates leaf damage.

In order to identify the sensitivity of transgenic cucumber plants to HT stress, the viability of root tips was assessed. The root tip viabilities of both WT and transgenic cucumber plants were decreased under the 43°C HT treatment, but there were more dead cells in WT plants relative to the transgenic plants (**Figure 5A**). Moreover, the heat resistance of transgenic plants was stronger than that of WT plants, and HTs affected the root viability of the plants.

The transgenic and WT plants were treated at 43°C for three days, after which their chlorophyll fluorescence parameters were measured. The chlorophyll fluorescence parameters of both were significantly affected, but all parameters of the WT plants were lower than those of the transgenic plants (**Table 1**). The damage to the photosynthesis systems of the WT plants was more serious than to that to the transgenic plants under HT stress (**Figure 5B**), suggesting overexpression of *CsCaM3* could increase heat-resistance of cucumber plants.

**Table 1:** Effects of HT stress on chlorophyll fluorescence parameters of transgenic and wild-type (WT) plants.

Material	*F*_v_/*F*_m_	Φ_PSII_	NPQ	*qP*	ETR
WT	0.732 ± 0.03a	0.521 ± 0.01a	0.618 ± 0.02a	0.913 ± 0.04a	0.812 ± 0.03a
OE-1	0.841 ± 0.02b	0.562 ± 0.01b	0.678 ± 0.02b	0.973 ± 0.03b	1.135 ± 0.02b
OE-2	0.813 ± 0.01b	0.573 ± 0.02b	0.752 ± 0.03c	0.964 ± 0.02b	1.143 ± 0.03b
OE-3	0.825 ± 0.04b	0.566 ± 0.01b	0.781 ± 0.02c	0.967 ± 0.02b	1.238 ± 0.04b

*Mean values are shown with ± standard errors. The lowercase letters “a, b, and c” indicate significant difference from wild-type (WT) plants at *P* < 0.05.*

### Detection of ROS, Chlorophyll Contents, Antioxidant Enzymes Activity, and MDA in Transgenic Plants Under HT Stress

**FIGURE 6:**
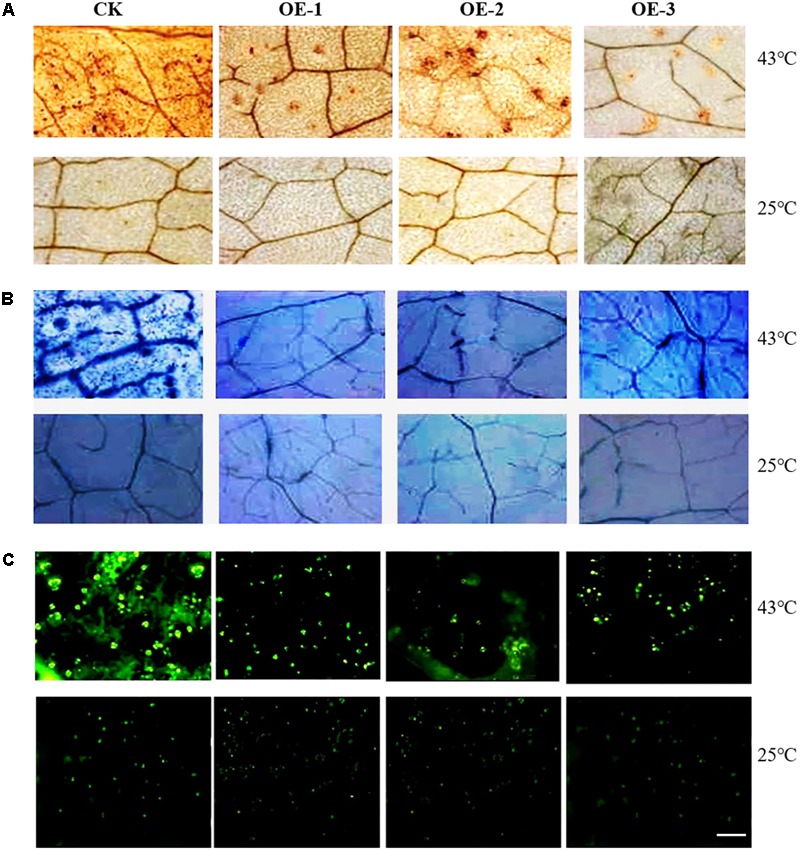
ROS detection of transgenic cucumber plant leaves under HT stress. **(A)** H_2_O_2_ was detected by staining with 3,3-diaminobenzidine (DAB). Brown spots indicate H_2_O_2_ accumulation in leaves. **(B)** Superoxide was detected by nitro blue tetrazolium (NBT) staining. Blue spots indicate the superoxide accumulation in leaves. **(C)** ROS was assayed with 2′,7′-dichloro dihydro fluorescein diacetate (DCFH-DA) staining, and green spots show the degree of overall ROS stress in leaves. Scale bar = 2.5 mm.

The contents of overall ROS, H_2_O_2_ and superoxide in the leaves of transgenic plants were lower than those of the WT plants, but under normal conditions, the ROS content of both the transgenic and WT did not significantly differ (**Figure 6**). Compared to the control plants, the chlorophyll contents of both the WT and transgenic plants were not remarkably different. However, the activities of SOD, POD, and CAT of the transgenic plants were significantly higher than those of the WT plants, and the MDA contents of WT plants were significantly higher than those of the transgenic plants (**Table 2**). These results again demonstrate that overexpression of *CsCaM3* in cucumber could increase the activities of antioxidant enzymes and decrease MDA content and oxidative stress damage.

**Table 2:** Chlorophyll contents, antioxidant enzymes activities, and MDA content in transgenic plants under HT stress treatment for 3 days.

	Chl (mg g^-1^ FW)	Chl a (mg g^-1^ FW)	Chl b (mg g^-1^ FW)	SOD (unit g^-1^ FW)	POD (unit g^-1^ FW min)	CAT (μmol mg^-1^ min)	MDA (mmol g^-1^ FW)
WT	4.17. ± 0.21a	2.45 ± 0.11a	1.12 ± 0.08a	38.2 ± 2.13a	128.4 ± 9.31a	1.31 ± 0.08a	0.97 ± 0.02a
OE-1	6.58 ± 0.31b	4.83 ± 0.15b	2.29 ± 0.05b	44.56 ± 2.33b	145.67 ± 8.43b	2.14 ± 0.03b	0.71 ± 0.02b
OE-2	6.37 ± 0.45b	4.94 ± 0.13b	2.34 ± 0.03b	45.71 ± 3.05b	147.23 ± 8.57b	2.24 ± 0.04b	0.75 ± 0.05b
OE-3	6.55 ± 0.43b	4.96 ± 0.11b	2.80 ± 0.06b	45.83 ± 2.46b	145.87 ± 7.94b	2.07 ± 0.07b	0.78 ± 0.06b

*Mean values are shown with standard error. The lowercase letters “a and b” indicate significant difference from wild-type (WT) at *P* < 0.05; Chl, chlorophyll; Chl a, chlorophyll a; Chl b, chlorophyll b; SOD, superoxide dismutase; POD, peroxidase; CAT, catalase; MDA, malondialdehyde.*

### Expression of Genes Related to Stress Tolerance and Chlorophyll Catabolism in Transgenic Plants Under HT Stress

**FIGURE 7:**
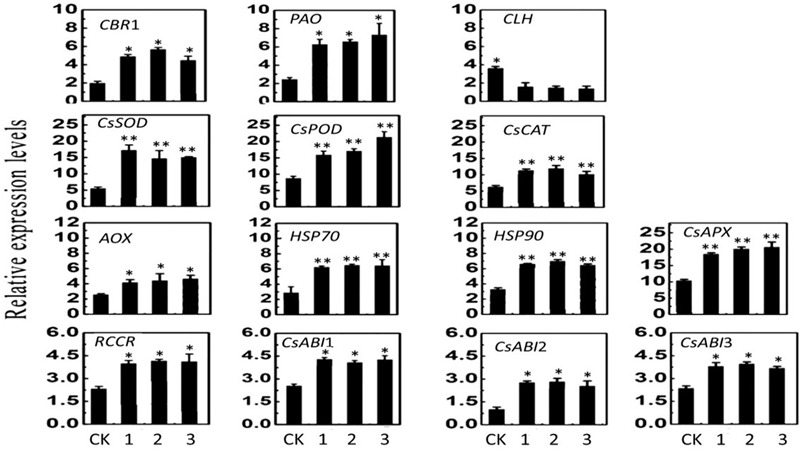
Expression of genes in transgenic and WT plants under 43°C-day/25°C-night temperature stress for 3 days in WT plants and transgenic OE-1, OE-2, and OE-3 plants (1, 2, and 3, respectively). Asterisks indicate significant differences from WT at ^∗^*P* < 0.05 and ^∗∗^*P* < 0.01.

The *CLH, CBR1, PAO*, and *RCCR* genes are related to the chlorophyll catabolic pathway in plants, and their expression reflects the process of photosynthesis. Therefore, the expression levels of these four genes were measured. The transcript levels of the four genes substantially differed between the WT and transgenic plants. The expressions of *CBR1, PAO*, and *RCCR* in transgenic plants were higher than those of WT plants, but the expression of *CLH* in transgenic plants was lower than that of the control plants under the 43°C temperature treatment for 3 days. At the same time, the expression levels of seven genes (*CsSOD, CsPOD, CsCAT, CscAPX, AOX, HSP70*, and *HSP90*) in the transgenic plants were markedly elevated and were higher than those of the WT plants. The expression levels of *CsABI1, CsABI2*, and *CsABI3*, which are involved in the ABA catabolic pathway in plants, were significantly increased compared with the WT plants (**Figure 7**). These results suggest that overexpression of *CsCaM* could affect the transcription of genes not only in the chlorophyll pathway, but also in the ABA catabolic pathway.

### Analysis of ABA Content and Stomatal Aperture

**FIGURE 8:**
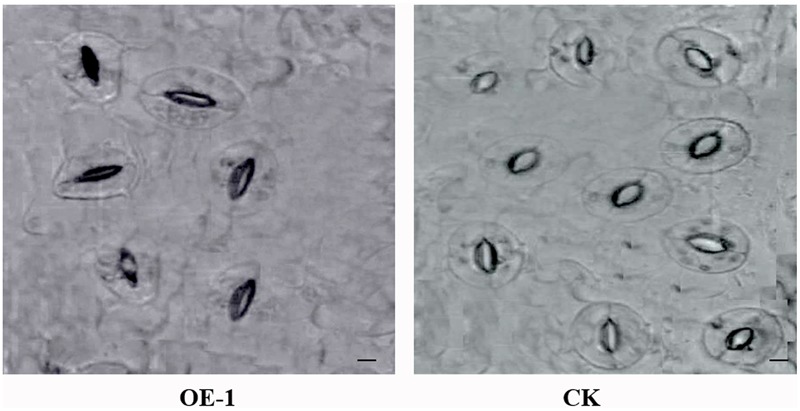
Images of stomata in transgenic and wild-type (WT) plants leaves under HT stress. OE-1 corresponds to a transgenic plant, and WT represents wild-type plants. Stomata were measured after 43°C HT treatment for 3 days. Scale bar = 20 μm.

After 3 days of the 43°C temperature treatment, the ABA content of the transgenic and WT plants was assayed. The ABA content of all transgenic plants was significantly higher than that of the WT plants (**Table 3**). On the other hand, the opening degree of stomata of WT plants leaves was greater than that of the transgenic plants under the temperature stress treatment (**Figure 8**).

**Table 3:** Contents of abscisic acid and size of stomatal aperture.

Material	ABA (ng g^-1^)	Stomatal size	
		Width (μm)	Length (μm)
WT	9.83 ± 0.41a	17.23 ± 1.02a	25.42 ± 1.13a
OE-1	15.76 ± 0.84b	11.38 ± 0.85b	18.33 ± 0.84b
OE-2	16.31 ± 0.72b	11.85 ± 0.91b	17.52 ± 0.35b
OE-3	15.92 ± 0.65b	10.65 ± 0.54b	18.57 ± 0.11b

*Mean values are shown with standard errors. The lowercase letters “a and b” indicate significant differences from wild-type (WT) at *P* < 0.05; OE-1, OE-2, and OE-3, transgenic plants.*

## Discussion

Plants have multiple pathways that allow them to physiologically adapt to environmental stress; HTs are a major stress factor affecting plant ecology and agricultural production (Mittler and Blumwald, 2010). CaM is a ubiquitous and conserved Ca^2+^-binding protein that regulates a vast array of Ca^2+^-sensitive biological events including Ca^2+^ transport, long-term intracellular signaling, and transcriptional regulation (Kortvely and Gulya, 2004). CaM is also involved in stress tolerance in plants (Kim et al., 2009; Singh et al., 2017). Overexpression of CaM in plants has been reported to improve the heat tolerance of plants (Larkindale and Knight, 2002; Li and Gong, 2009; Zhang et al., 2009), but there is a lack of reports that have been published about the transformation of CaM into cucumber. In our study, *CsCaM3* had high similarity to CaM genes that are related to stress-regulation in other plants and was verified to improve heat tolerance in cucumber through overexpression. Our results indicated that overexpression of *CsCaM* in cucumber could protect against oxidative damage and photosynthesis system damage under HT stress (**Figure 4**), concurring with results obtained in *Arabidopsis* (Larkindale and Knight, 2002) and tobacco (Li and Gong, 2009).

Subcellular localization revealed that *CsCaM3* proteins were located in the membranes and nuclei of cells (**Figure 3**), reflecting similar results in pea (Dauwalder et al., 1986), *Petunia* (Rodriguez-Concepcion et al., 1999; VanDer Luit et al., 1999). CaM is also localized in peroxisomes, plastids, mitochondria, and the extracellular matrix (Jarrett et al., 1982; Roberts et al., 1983; Ma et al., 1999; Reddy et al., 2002; Yang and Poovaiah, 2002), which is consistent with the location of majority of its target proteins that in the plasma, membrane, or nucleus (Virdi et al., 2015). Target proteins affected by *CsCaM* are still unknown in this study and should be isolated and identified in the future.

The expression of *CsCaM3* differed among plant organs and tissues (**Figure 2A**), and could be induced by HT stress and ABA application (**Figures 2B,C**), concurring with results reported by other researchers (Liu et al., 2005; Zhang et al., 2013; Munir et al., 2016). However, *CaM3* transcripts in tall fescue decreased under HTs (Wang and Huang, 2017); thus, different plants may have different stress-tolerance regulation mechanisms. It was reported that calcium-activated CaM (Ca^2+^-CaM) was directly involved in the heat stress signal transduction pathway in wheat (*Triticum aestivum*) and up-regulated heat shock proteins (HSPs; Liu et al., 2003). HSPs (such as HSP18, HSP70, HSP90, and HSP101) are known to be positive regulators of heat tolerance in various plant species (Zhou and Abaraha, 2007). Some reports have shown that CaM interacts with HSP90 and HSP70, of which steady state levels are regulated through Ca^2+^-CaM pathways (Sun et al., 2000; Virdi et al., 2011). And in our research, expression levels of *HSP70* and *HSP90* in transgenic cucumber plants were significantly higher compared with WT plants (**Figure 7**), and the way in which *CsCaM3* interacts with *HSP70* and *HSP90* remains to be identified in the future. The complex crosstalk between signaling molecules, HSPs, and partner proteins appears to be critical for heat stress responses (Wang and Huang, 2017).

Under adversity, plants will produce ROS, inducing oxidative stress, which damages cell membrane system. To counteract the deleterious effects of ROS, a complex antioxidative defense system evolved in plants, which includes antioxidant enzymes such as SOD, POD, CAT, and so on. Through the determination of the activity of antioxidant enzymes, the strength of stress resistance of plants can be identified (Suzuki et al., 2011; Gupta et al., 2013). H_2_O_2_ is one of the key elements in the ROS signaling pathway and was found to induce plant heat tolerance through exogenous application (Larkindale and Huang, 2005). H_2_O_2_ is also known to be involved in the early phase of heat stress and is required for effective expression of *HSPs* and *HSFs* in *Arabidopsis* and rice (*Oryza sativa*; Volkov et al., 2006; Wang et al., 2009). In our data, under HT stress, the contents of H_2_O_2_, superoxide, and the overall ROS in WT plants were higher in comparison with transgenic plants (**Figure 6**). In the current research, the antioxidant enzyme activities of SOD, POD, and CAT were all higher in transgenic plants, and the MDA content of transgenic plants was also lower compared with WT plants (**Table 2**), which may indicate increased oxidative stress tolerance in transgenic plants. At the same time, the expression levels of genes related to oxidative stress (such as *CsSOD, CsPOD, CsCAT, CsAPX*, and *AOX*) were all higher in transgenic plants (**Figure 7**); similar results were observed by other researchers (Kolupaev et al., 2008; Xu, 2010). For example, the expression of *SPCAT* in sweet potato was regulated by *SPCAM* (Afiyanti and Chen, 2014). However, a continuous influx of Ca^2+^ is required for activity associated with the production of H_2_O_2_ from NADPH, which is catalyzed by NADPH oxidase (Keller et al., 1998). The activity of NAD kinase is also adjusted through Ca^2+^-CaM (Harding et al., 1997), which is activated by H_2_O_2_ (Pei et al., 2000). These studies have elucidated the complex mechanisms of feedback modulation of different enzymes through Ca^2+^ and CaM, which may enable plants to maintain H_2_O_2_ homeostasis, despite stress-induced damage (Virdi et al., 2015). In our research, the above-mentioned genes related to heat stress all had active responses that included the overexpression of *CsCaM3*, but the mechanism through which overexpression of *CsCaM3* induced the expression of those genes remains unclear.

Enhanced ABA content in plants can improve stress tolerance (Larkindale and Knight, 2002). In our study, the ABA content of the transgenic plants was much higher relative to WT plants (**Table 3**), and the expression of *CsABI1, CsABI2*, and *CsABI3* in the ABA catabolic pathway was also upregulated under HTs (**Figure 7**); these genes might promote an increase in ABA content. We inferred that overexpression of *CsCaM* in cucumber increases the heat stress response of plants via the ABA signal pathway, but the regulation mechanism remains to be clarified. Under HT stress, the opening degree of stomata of transgenic plants leaves was lower than that of the control plants (**Table 3**). Because stomatal dynamic variation is a crucial ABA-regulated process, we deduced that high levels of ABA might make stomata close more quickly, as has been observed in Chinese kale (Zhu et al., 2016).

*CLH* is a key gene that regulates chlorophyll catabolism. The transcription of *CLH* in transgenic plants in the present study was significantly lower than that in WT plants under HTs (**Figure 7**), but the total chlorophyll, chlorophyll a, and chlorophyll b contents of the transgenic plant leaves were significantly higher than those of WT plants (**Table 2**). These results suggested that overexpression of *CsCaM3* may downregulate the transcription of *CLH* to reduce chlorophyll degradation under HTs. *CBR1, PAO*, and *RCCR* are also involved in the chlorophyll catabolic pathway. Chlorophyll b reductase (CBR) is a key enzyme for the transformation of chlorophyll b into chlorophyll a. Pheophorbide a is catalytically degraded to red chl catabolites (RCCs) by pheophorbide a oxygenase (PAO), and red chlorophyll catabolite reductase (RCCR) plays a key role for the conversion of RCCs into non-fluorescent chlorophyll catabolites (NCCs; Vezitskii and Shcherbakov, 2002; Pružinská et al., 2007). In our study, under HTs, the expression levels of *CBR1, PAO*, and *RCCR* in transgenic plants were markedly higher than those in WT plants (**Figure 7**), which may enhance the conversion of chlorophyll b into chlorophyll a. Chlorophyll b can enter the degradation pathway, and only after being converted into chlorophyll a can it be further metabolized (Hortensteiner and Krautler, 2011). Mutations of *PAO* and *RCCR* can be further metabolized and lead to an increase in degraded chlorophyll intermediates, thus accelerating cell death (Ginsburg and Matile, 1993; Ginsburg et al., 1994). We infer that high expression levels of *PAO* and *RCCR* can decrease the accumulation of potentially phototoxic chlorophyll intermediates and thus prevent them from injuring plants. The mechanism by which *CsCaM* regulates the expression of these genes under HT stress is a subject for future investigations.

## Author Contributions

BC contributed to designing the experiments. BC, BY, HZ, and RD performed the experiments, collected and analyzed the data. BC, JL, and CC contributed to data interpretation and preparation of the manuscript. All authors read and approved the final manuscript.

## Conflict of Interest Statement

The authors declare that the research was conducted in the absence of any commercial or financial relationships that could be construed as a potential conflict of interest.

## Footnotes

1 http://cucumber.genomics.org.cn

## Supplementary Material

FIGURE S1 Diagram of the pBI-*CsCaM3* expression cassette transformation vector.

FIGURE S2 The overexpression of *CsCaM3* in transgenic cucumber plants. Wild-type (WT) plants are shown beside the transgenic plants (OE-1, OE-2, OE-3, and OE-4).

TABLE S1 Primer sequences for qRT-PCR.
