# Supplementary figures and images for: Overexpression of CsCaM3 Improves High Temperature Tolerance in Cucumber
Source: Front Plant Sci. 2018 Jun 12;9:797. doi: 10.3389/fpls.2018.00797 (PMC6006952; doi:10.3389/fpls.2018.00797)

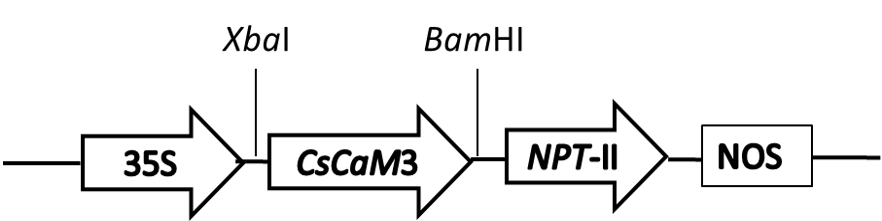

Supplement: FIGURE S1 — Diagram of the pBI-CsCaM3 expression cassette transformation vector. [file Image_1.TIF]

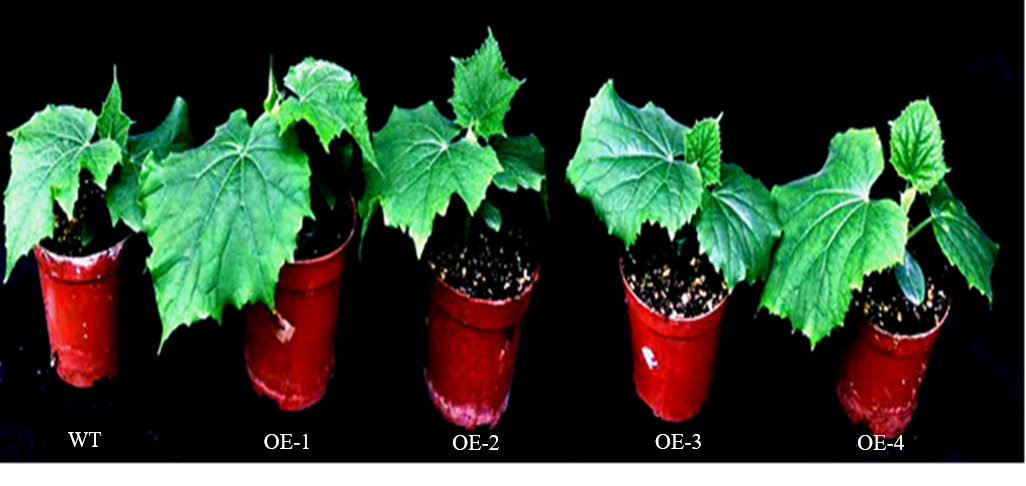

Supplement: FIGURE S2 — The overexpression of CsCaM3 in transgenic cucumber plants. Wild-type (WT) plants are shown beside the transgenic plants (OE-1, OE-2, OE-3, and OE-4). [file Image_2.TIF]
