# Supplementary material for: Overexpression of CsCaM3 Improves High Temperature Tolerance in Cucumber
Source: Front Plant Sci. 2018 Jun 12;9:797. doi: 10.3389/fpls.2018.00797 (PMC6006952; doi:10.3389/fpls.2018.00797)
Supplement: TABLE S1 — Primer sequences for qRT-PCR. [file Table_1.docx]

**Supplementary materials**

**Table S1** Primer sequences for qRT-PCR

| Gene | Forward primer | Reverse primer | gene number |
| --- | --- | --- | --- |
| *CsCaM3* | ATGGCGGATCAGCTCACCGA | CCTTAGCCATCATGACCTTAAC | XM_011655459 |
| *CsSOD* | GAGCAATCAGGGAGTCAGT | TACCATCATCACCAGCAAC | 101231664 |
| *CsPOD* | TTCTTGCCCTTCAGGTTGT | CTCCGATTGATTTGTTCCA | 101228793 |
| *CsCAT* | AACAACACCGCCGTAATGT | ATGACGGGGGTTTGGACG | Csa6M356990 |
| *CscAPX* | ATGGCACTCTGCTGGAAC | GTCTGCATATGAGAGGATGG | Csa1M479610 |
| *CsABI1* | AAGATTTGGGATTTGGTGA | TACGGTAAGATGATGATGACG | 101229497 |
| *CsABI2* | CTGTCCACTTCTCCCACC | ACTCCATACGCCGTCTTT | 101230352 |
| *CsABI3* | ACTGAGATTGGCAAAGATA | GACTAATGGTCCTGGGTAA | 101230126 |
| *AOX* | TCATCATCACCGAACTTACA | GAATCCACCATCCGACAA | DQ641114 |
| *HSP70* | GGCTGAGGCAGATGAGTTCGAGGA | GGCCAGCACCGCCGCTACCA | 101210094 |
| *HSP90* | AAAGAAGAAGAAAATCAAGGAAGT | ACTGAGAAGTGTTTCACGGCTAA | 101221871 |
| *PAO* | GGAATGAGGGTTCGTCTA | CAAAGCAGGGTCCAAGTC | 101217499 |
| *CLH* | TATGGTGGAACTGGTATTG | TTAACTGTCGCTTTCTTCT | 101206766 |
| *RCCR* | GACAGATGTGGAGTATTATGAG | GAGAAGATGGAAGAGCAGAA | 101223133 |
| *CBR1* | TCAGGAGTTTCAGTCTTTC | GTGGATACGGAATTGGAA | 101222986 |
| *Actin* | AGAGATGGCTGGAATAGAAC | CTGGTGATGGTGTGAGTC | DQ641117 |
